# Prognosis and immune response of a cuproptosis-related lncRNA signature in low grade glioma

**DOI:** 10.3389/fgene.2022.975419

**Published:** 2022-10-21

**Authors:** Yifan Xu, Chao Wang, Shifang Li, Han Zhou, Yugong Feng

**Affiliations:** Department of Neurosurgery, Qingdao Affiliated Hospital, Qingdao, China

**Keywords:** low grade glioma, lncRNA, cuproptosis, immune therapy, bioinformatics

## Abstract

Cuproptosis is a newly discovered new mechanism of programmed cell death, and its unique pathway to regulate cell death is thought to have a unique role in understanding cancer progression and guiding cancer therapy. However, this regulation has not been studied in low grade glioma (LGG) at present. In this study, data on low grade glioma patients were downloaded from the TCGA database. We screened the genes related to cuproptosis from the published papers and confirmed the lncRNAs related to them. We applied univariate/multivariate, and LASSO regression algorithms, finally identified 11 lncRNAs for constructing prognosis prediction models, and constructed a risk scoring model. The reliability and validity test of the model indicated that the model could well distinguish the prognosis and survival of LGG patients. Furthermore, the analyses of immunotherapy, immune microenvironment, as well as functional enrichment were also performed. Finally, we verified the expression of these six prognostic key lncRNAs using real-time polymerase chain reaction (RT-PCR). In conclusion, this study is the first analysis based on cuproptosis-related lncRNAs in LGG and aims to open up new directions for LGG therapy.

## Introduction

Gliomas are the most common and long-lasting malignant primary brain tumors (Jiang et al., 2016), including low-grade gliomas (LGGs) and glioblastomas (GBMs). LGG, a diffuse low- and intermediate-grade glioma (WHO grades II and III), is a rare glioma that can be divided into different subtypes based on IDH1/2 mutation status and the presence of 1p19q co-deletion (Brat et al., 2015). In general, LGG is more indolent than glioblastoma (GBM) (WHO grade IV) (Louis et al., 2007; Ceccarelli et al., 2016; Ostrom et al., 2018). However, compared with other subtypes, the IDH1/2wt subtype is more aggressive, difficult to completely resect with surgical treatment, and the residual tumor can rapidly develop into GBM, making subsequent treatment difficult (Suzuki et al., 2015). In LGG, different IDH1/2 mutation statuses also resulted in different median survival, ranging from 1 year to 8 years (Brat et al., 2015). The high heterogeneity of LGG leads to difficult histological diagnosis prediction, high uncertainty, and unpredictable prognosis, so there are an urgent need to discover and further study effective biomarkers (Lapointe et al., 2018).

In 2012, Columbia University discovered a new type of programmed cell death, ferroptosis, which is completely different from apoptosis, programmed death, and pyroptosis in its mode of action and molecular mechanism. A type of programmed cell death induced by excessive accumulation of lipid peroxides (Dixon et al., 2012). In 2019, Xin et al. introduced the mechanism of ferroptosis in cancer in detail, pointing out the potential application of ferroptosis in systemic therapy, radiotherapy, and immunotherapy (Chen et al., 2021). Like iron, copper also plays an important role in the regulation of biological activity. Excessive copper accumulation induces apoptosis or necrosis, leading to cell death. In March 2022, the Harvard-MIT Broad Institute revealed the mechanism of cuproptosis for the first time. Unlike other mechanisms, cuproptosis is through direct copper ions and fatty acylation components of the tricarboxylic acid cycle (TCA) in mitochondrial respiration (Ruiz et al., 2021). Binding occurs, leading to fatty acylated protein aggregation and subsequent iron-sulfur clusterin downregulation, leading to proteotoxic stress and eventual cell death (Tsvetkov et al., 2022). Although elesclomol, a copper-supported small molecule anticancer drug, did not significantly improve the survival of tumor patients in clinical trials, posthoc analysis showed that this class of drugs has some effect on inhibiting tumor cells that rely on mitochondrial production capacity (O'Day et al., 2013). Target proteins encoding elesclomol were shown to share genes that promote cuproptosis (Tsvetkov et al., 2022).

Long non-coding RNAs (lncRNAs) are a class of RNAs longer than 200 nucleotides without the capacity to encode for protein. They have important functions in transcriptional silencing, transcriptional activation, chromosome modification, and intranuclear transport and perform important regulatory capabilities (Wang et al., 2021). A large number of studies have proved that lncRNAs are closely related to the occurrence, development, and prognosis of cancer. For example, lncRNAsMALAT1 can be used as a therapeutic target for cancer treatment (Amodio et al., 2018). In LGG, Xu and his team found that LINC00941 could predict the prognosis of LGG (Xu et al., 2021). LncRNA LINC00336 was found to have an inhibitory role in ferroptosis (Wang et al., 2019). Overexpression of lncRNA NEAT1 can increase ferroptosis, thereby increasing the antitumor activity of erastin and RSL3. However, the regulation of lncRNAs in cuproptosis remains unclear.

As the first biological study to integrate lncRNAs with cuproptosis in LGG, we aim to establish a new signature of cuproptosis-related lncRNAs to predict the prognosis of LGG patients and to evaluate the efficacy of these lncRNAs. Based on the TCGA database, we finally screened 11 of them and constructed a risk model to predict OS in LGG patients. We then divided LGG patients into two groups (high-risk and low-risk) based on mean-risk scores. Through the analysis, we found that high-risk groups had better responses to immunotherapy, suggesting that the model could serve as a potential biomarker to predict the prognosis of LGG patients. We hope that our study will improve the accuracy of prognosis prediction and elucidate the possible mechanism of cuproptosis-related lncRNAs in LGG.

## Methods

### Data acquisition

RNA sequence transcriptome data, clinical information, and mutation data of LGG were downloaded from the TCGA database (https://cancergenome.nih.gov/). Ten cuproptosis-associated genes including FDX1, LIAS, LIPT1, DLD, DLAT, PDHA1, PDHB, MTF1, GLS, and CDKN2A were acquired from the literature (Kahlson and Dixon, 2022). We excluded samples (0/<30 OS values) for reducing statistical bias and finally obtained 479 LGG patients for subsequent bioinformatics analysis.

### Cuproptosis-related LncRNAs

Screening cuproptosis-related lncRNAs based on Pearson’s correlation analysis and thus identifying 677 cuproptosis-related lncRNAs. The process followed the rule of |*Pearson R*| > 0.4 and *p* < 0.001.

### Construction of the cuproptosis-related LncRNA risk signature

We randomly divided the 479 LGG samples into the training set (*n* = 240) and the testing set (*n* = 239). The baseline characteristics of the training set and the testing set are shown in Supplementary Table S1, and the two datasets are consistent in clinical characteristics (*p* > 0.05). The prognostic values of each lncRNA were first evaluated using univariate Cox regression analysis. The least absolute shrinkage and selection operator (LASSO) Cox regression were performed to reduce the dimension of high-latitude data using R package “glmnet”. Ten-fold cross-validation was employed to avoid the overfitting problem and select the penalty parameter (*λ*) according to the minimum criteria. Multivariate Cox regression analysis was then performed to identify the final candidates involved in the risk signature. The testing set and the entire set were used to validate its accuracy of it. The risk score for each LGG patient was counted with the following algorithm: risk score = expression of a lncRNA [1] × corresponding coefficient [1] + expression of a lncRNA [2] × corresponding coefficient [2] + expression of a lncRNA [n] × corresponding coefficient [n]. Additionally, we classified the LGG patients into two groups based on their risk scores: low-risk group and high-risk group.

### Assessment of the prediction ability of risk signature

We used Kaplan-Meier analysis to test the accuracy of the established model using R packages “survival” and “survminer”. Furthermore, we conducted principal component analysis (PCA) (Ringnér, 2008) and *t*-distributed stochastic neighbor embedding (*t*-SNE) (Belkina et al., 2019) analysis to visualize high-risk and low-risk groups as well as test the performance of the established model.

### Independent prognostic factor analysis and nomogram

To determine whether the risk score was superior to other clinical traits, an independent prognostic factor analysis utilizing univariate and multivariate Cox regression was conducted in R with the package “survival”. Using R package “RMS”, we established a nomogram integrated risk scores as well as other clinicopathological characteristics (age, gender) to better predict the 1-, 3-, and 5-year OS. Besides, we applied calibration curve analysis to examine the reliability of the established nomogram. The prediction accuracy was examined by using the receiver operating characteristic (ROC) curves and conformance index (C-index).

### Analysis of tumor microenvironment

We applied the estimate algorithm to calculate each patient’s immune score, stromal score, and estimate score using the R package “ESTIMATE” (Chakraborty and Hossain, 2018). Furthermore, we conducted ssGSEA (Shen et al., 2019) and CIBERSORT (Newman et al., 2015) algorithms to quantify the infiltration of 22 immune cells and immune functions in the tumor immune microenvironment (TME).

### Analysis of somatic mutation

We applied VarScan software to process the “mask somatic mutation” data from the TCGA database (Wormald et al., 2018). The tumor mutation burdens (TMBs) were measured using the R package “maftools”. For dividing patients into high and low TMB groups, the median TMB score was used as a cut-off value.

### Analysis of drug sensitivity

The IC50 was calculated using the R package “pRRophetic”, and the chemotherapeutic medications were obtained from the Genomics of Drug Sensitivity in Cancer (GDSC) database (Yang et al., 2013). The IC50 of high-risk and low-risk groups was then compared by the Wilcoxon sign rank test.

### Quantitative real-time PCR analysis of long non-coding RNAs in glioma tissues

All tissue samples were collected from the Neurosurgery Department of Qingdao University Affiliated Hospital, which was approved by the Medical Ethics Committee of the hospital. All specimens were stored in liquid nitrogen, and all patients provided informed consent. From July 2022 to August 2022, four low grade glioma tissue samples and four non-tumor brain tissues were obtained. Total RNA was isolated using TRIzol reagent, quantitative real-time fluorescence quantitative PCR(qRT-PCR) was performed by using primers (Beijing Genomics institution) and premix (SYBR Master Mix). Data were analyzed using the 2^−ΔΔCt^ method with each test performed in triplicate. The primer sequence is shown in Supplementary Table S1.

### Functional analysis and mRNA-lncRNA Co-Expression network

We applied the “limma” R package to distinguish the differentially expressed genes (DEGs) in the subgroups following criteria (|log2-fold change (FC)| ≥ 1, *p*-value < 0.05). GO and KEGG enrichment analyses were applied using the package “clusterProfiler” in R. We performed a GSEA analysis using the GSEA software (http://www.gesa-msigdb.org/gsea/index,jsp) to further screen the difference of functional pathways between high-risk and low-risk groups. The network among lncRNAs, mRNAs, and risk types was visualized by Cytoscape. Furthermore, the correlation between cuproptosis-related lncRNAs, cuproptosis associated genes, and factors with risk or protection was analyzed using the R package “ggalluvial”.

## Results

### Acquisition of cuproptosis-associated lncRNAs in LGG

**FIGURE 1:**
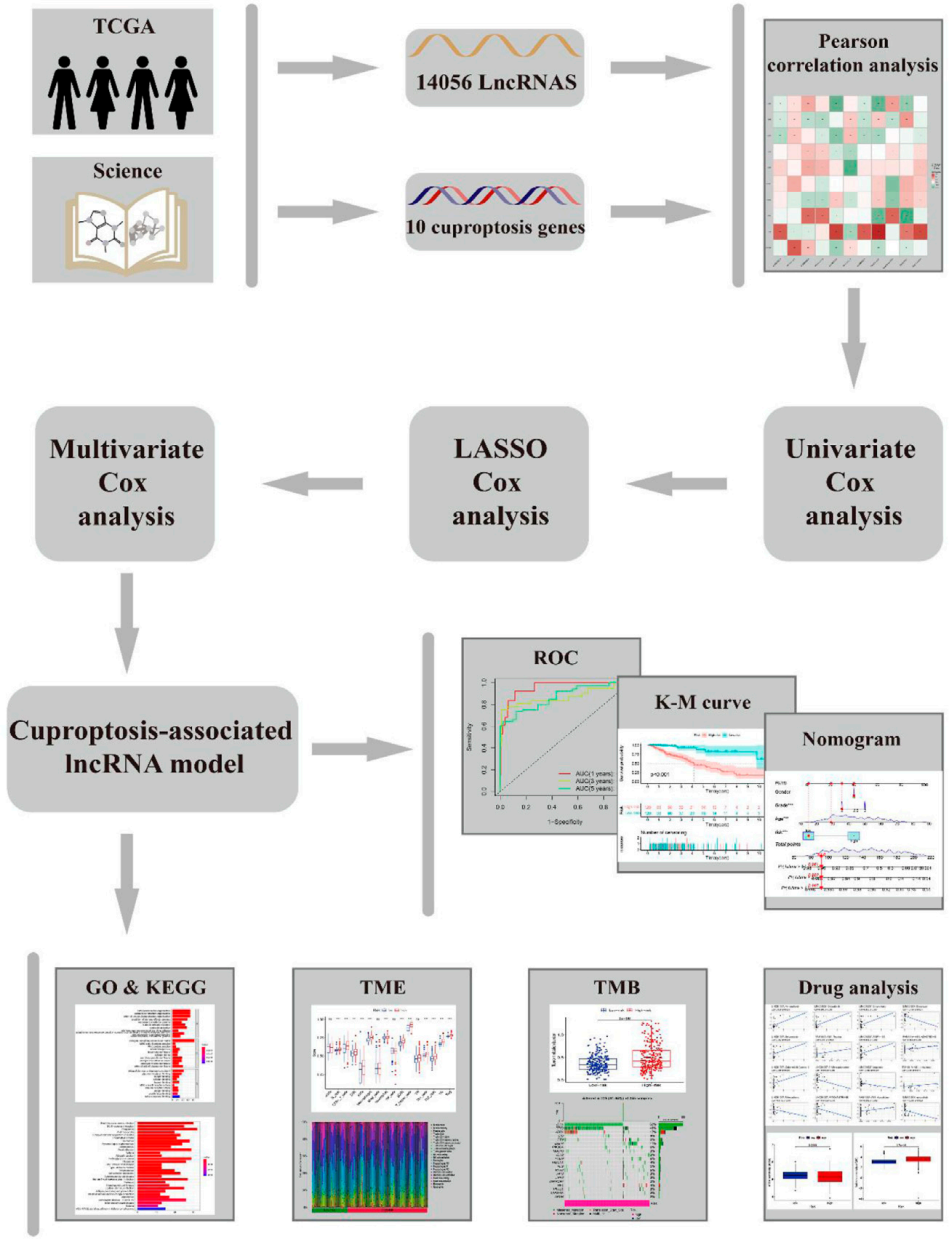
Flow chart.

**FIGURE 2:**
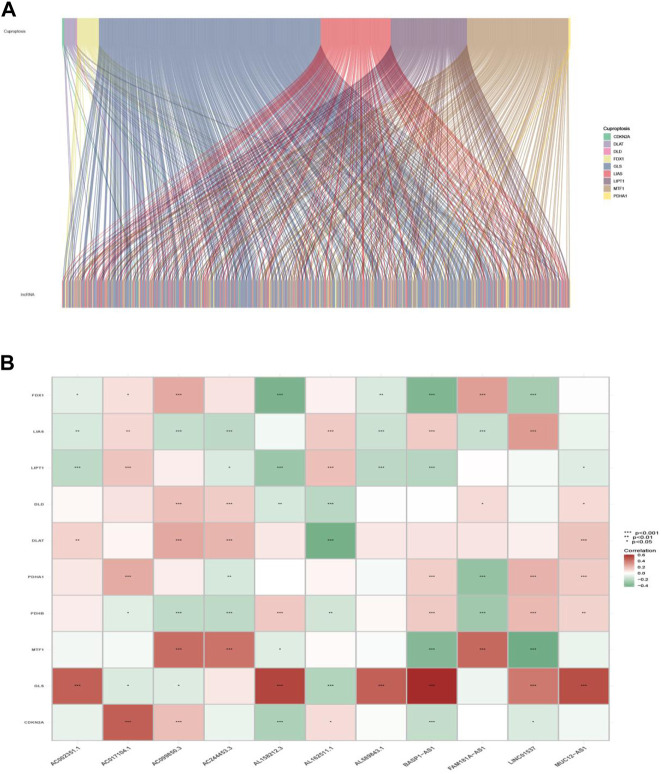
Cuproptosis-related gene and lncRNA profile in this study. **(A)** Sankey relation diagram for cuproptosis genes and lncRNAs. **(B)** Heatmap for the correlations between cuproptosis genes and lncRNAs.

The workflow of this study is shown in Figure 1. Clinical features of the final 479 samples shown in Supplementary Table S2, as well as RNA profiles and somatic mutation data, were gathered from the TCGA database. Subsequently, we retrieved the expression profiles of 10 cuproptosis-associated genes and 14,056 lncRNAs, while a total of 677 cuproptosis-associated lncRNAs were acquired among lncRNAs and cuproptosis genes based on the linkage intensity of (|*Pearson R*| > 0.4 and *p* < 0.001) (Supplementary Table S3 **)**. The Sankey diagram in Figure 2A interpreted the correlation relationship between cuproptosis genes and cuproptosis-associated lncRNAs. Also, the outcome performed by Pearson’s correlation analysis was visualized in Figure 2B, with more details recorded in Supplementary Table S4.

### Developing the cuproptosis-related LncRNAs risk model

**FIGURE 3:**
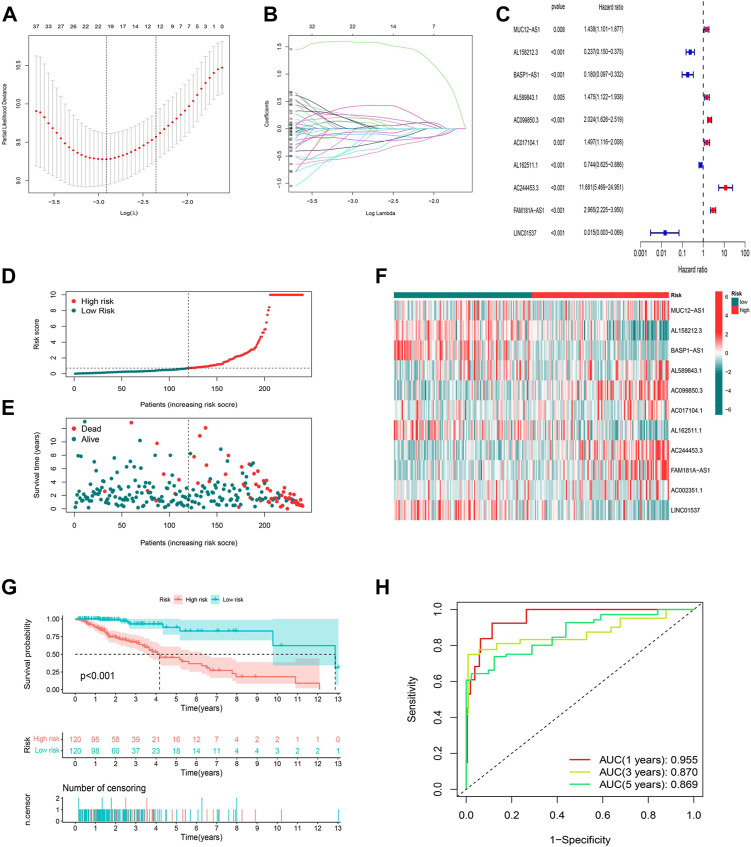
Construction and validation of the prognostic model in the TCGA training set. **(A)** The LASSO coefficient profile of cuproptosis-related lncRNAs. **(B)** The 10-fold cross-validation for variable selection in the LASSO model. **(C)** Multivariate Cox regression analysis showed six independent prognostic lncRNAs. **(D)** Distribution of cuproptosis-related lncRNAs model-based risk score for the training set. **(E)** Different patterns of survival status and survival time between the high-risk and low-risk groups for the training set. **(F)** The clustering analysis heatmap shows the expression standards of the six prognostic lncRNAs for each patient in the training set. **(G)** Kaplan-Meier survival curves of the OS of high-risk and low-risk patients in the training set. **(H)** 1-, 3-, and 5-year ROC curve of the risk model.

To identify the ideal risk signature to guide the prognosis in patients with LGG, the univariate Cox regression analysis was conducted to screen for 297 cuproptosis-related lncRNAs associated with OS in concert with training set information (Supplementary Table S5). Furthermore, 19 cuproptosis-related lncRNAs were identified using LASSO Cox regression analysis (Figures 3A,B). Finally, the risk model was developed with 11 cuproptosis-associated lncRNAs which were determined by multivariate Cox regression analysis (Figure 3C).

The risk score was produced with the formula: risk score = expression of MUC12-AS1 × 0.382030727494924 + expression of AL158212.3 × -1.39920798375446 + expression of BASP1-AS1 × -1.9645994664291 + expression of AL589843.1 × 0.758072939276948 + expression of AC099850.3 × 0.311101322216435 + expression of AC017104.1 × 0.654061856724554 + expression of AL162511.1 × -0.688335466581498 + expression of AC244453.3 × 2.05053269718406 + expression of FAM181A-AS1 × 0.515445156843301 + expression of AC002351.1 × 0.556137908535174 + expression of LINC01537 × -1.84136636638082.

In this way, we were able to quantify the risk scores of all LGG patients and identify high- and low-risk groups based on the median risk score as the cutoff value in the training sets. All of the figures (Figures 3D–F) in the training set suggested that the higher the risk score, the worse the prognosis for patients with LGG, and this was again illustrated by the difference (*p* < 0.001) in OS between high- and low-risk groups in the K-M analysis curve (Figure 3G). We further conducted a time-dependent ROC curve analysis in the training set to compare the prediction performance of the risk signature at different time points. The AUCs for 1-, 3-, and 5-year were 0.955, 0.870, and 0.869, respectively, implying that the model based on cuproptosis-associated lncRNAs has good accuracy (Figure 3H).

### Validation of cuproptosis-related lncRNA risk model

**FIGURE 4:**
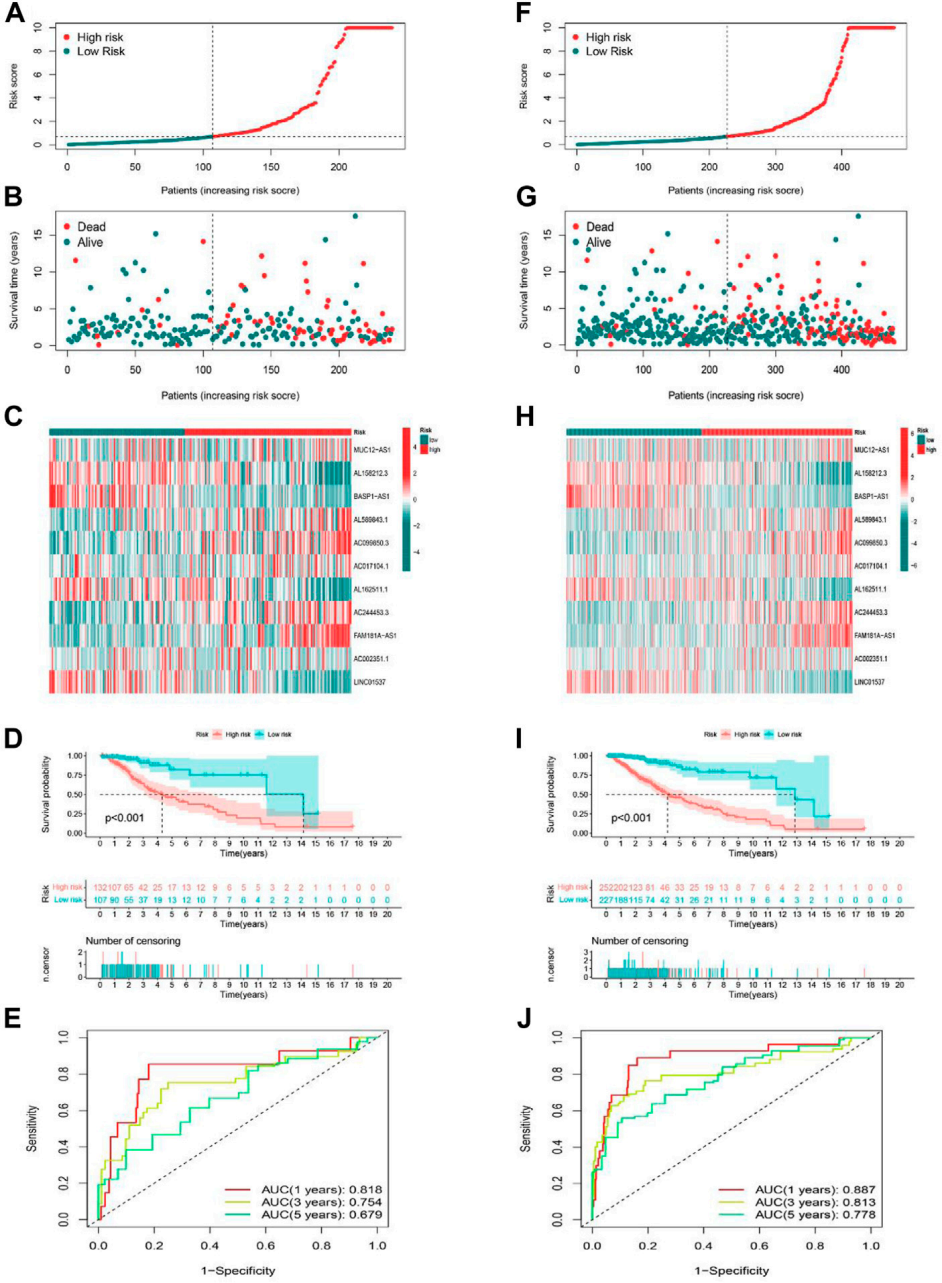
Prognostic value of the risk model of the 11 lncRNAs in the testing and entire sets. **(A–C)** The distributions of the risk scores, survival status, and the expression heatmap for 11 hub lncRNAs in the testing set. **(D)** Kaplan-Meier survival curves of the OS of patients in the high-risk and low-risk groups in the testing set. **(E)** The AUCs of testing are set for 1-, 3-, and 5-year OS rates. **(F–H)** The distributions of the risk scores, survival status, and the expression heatmap for 11 hub lncRNAs in the entire set. **(I)** Kaplan-Meier survival curves of the OS of patients in the high-risk and low-risk groups in the entire set. **(J)** The AUCs of the entire set for 1-, 3-, and 5-year OS rates.

The predictive ability of the risk signature was validated in both the testing set as well as the entire set. We used the same formula to calculate the risk scores of LGG patients in the training and entire set, and patients were also divided into high-risk as well as low-risk groups according to the same cutoff value. The distribution of risk scores, the pattern of survival status, and survival time, as well as the expression of the 11 cuproptosis-related lncRNAs in the testing set (Figures 4A–C) and the entire set (Figures 4F–H), suggested the same trend as aforementioned studies. K-M survival analysis also presented a substantial difference in OS among the low-risk and high-risk groups based on the testing set (Figure 4D **,** *p* < 0.001) and the entire set (Figure 4I **,** *p* < 0.001). Additionally, the AUCs for 1-, 3-, 5-year OS rates were 0.818, 0.754, 0.679, respectively based on the testing set (Figure 4E), and 0.887, 0.813, 0.778 based on the entire set (Figure 4J). These results demonstrated that the risk model based on 11 cuproptosis-related lncRNAs is stable and reliable.

### Exploration of the risk signature in a spatial arrangement

**FIGURE 5:**
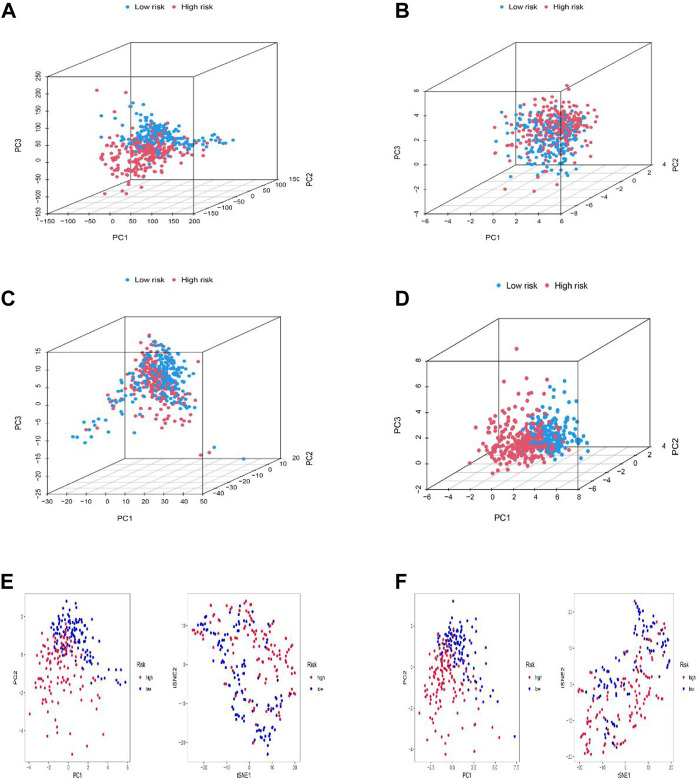
Principal component analysis. **(A–D)** PCA analysis between the high and low-risk based on the whole genome expression set **(A)**, 10 cuproptosis-related genes **(B)**, 1272 cuproptosis-related lncRNAs **(C)**, and risk model classified by the expression profiles of the six cuproptosis-related lncRNAs **(D)**. **(E,F)** PCA and t-SNE analyses between the high and low-risk groups in the training **(E)** and testing sets **(F)**.

PCA, as well as t-SNE analyses, were both utilized to probe the relationship between two subgroups in terms of spatial distribution patterns. PCA analysis was first performed at the RNA transcriptome level, including a whole gene expression profile based on the TCGA-LGG database, 10 cuproptosis-associated genes, 677 cuproptosis-related lncRNAs, and the risk model based on 11 cuproptosis-related lncRNAs (Figures 5A–D). Furthermore, an equivalent performance was also well represented in the training and testing sets based on analyses of PCA and t-SNE (Figures 5E,F). The above results revealed the excellent performance of our constructed prognostic risk model based on 11 cuproptosis-associated lncRNAs in distinguishing LGG patients into high- and low-risk groups, again demonstrating the accuracy of the model.

### Nomogram and correlation of the risk model with clinical characteristics

**FIGURE 6:**
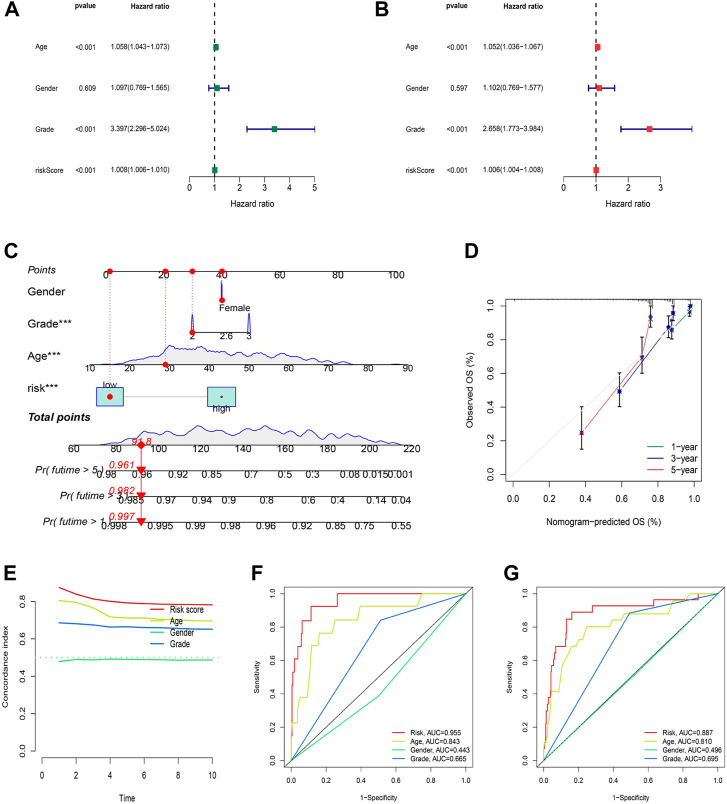
Construction and validation of the nomogram. **(A)** Univariate regression analysis in the testing set. **(B)** Multivariate Cox regression analysis in the testing set. **(C–D)** Uni/multivariate Cox regression in the training set. **(E)** The monogram. **(F)** The prediction accuracy of the nomogram. **(G)** The concordance index analysis of nomogram.

Independent analysis was conducted to judge whether the risk score based on cuproptosis-associated lncRNAs could be a prognostic factor for LGG. As a result, univariate and multivariate Cox regression analyses were performed to fulfill the requirement for identifying independence factors. As shown by univariate Cox analysis (Figure 6A), the risk score was substantially correlated with OS in LGG patients, as indicated by the hazard ratio (HR) and 95% confidence interval (CI) of 1.008 and 1.006–1.010 (*p* < 0.001). Likewise, in the multivariate Cox analysis (Figure 6B), the risk score still had a significant impact on prognosis and survival after adjusting for other confounding factors with the HR and 95% CI being 1.006 and 1.004–1.008 (*p* < 0.001). As nomograms is widely used to predict the survival and prognosis of cancer patients. Then we established a nomogram using the risk score and clinicopathological features such as gender, grade, and age to better estimate the 1-, three- and 5-year survival rate for LGG patients (Figure 6C). The calibration curve showed a great fit of 1-, 3-, and 5-year OS predicted by the nomogram compared to the actual observed OS (Figure 6D). In addition, the largest area of the risk score in the C-index meant that the model had considerable confidence in determining the prognosis of LGG patients (Figure 6E). Similarly, the risk score also occupied the maximum area of the ROC curves (Figures 6F–H) for the training (AUCs = 0.955), testing (AUCs = 0.818), and entire sets (AUCs = 0.887), indicating that the model was highly discriminatory.

### Functional enrichment analysis

**FIGURE 7:**
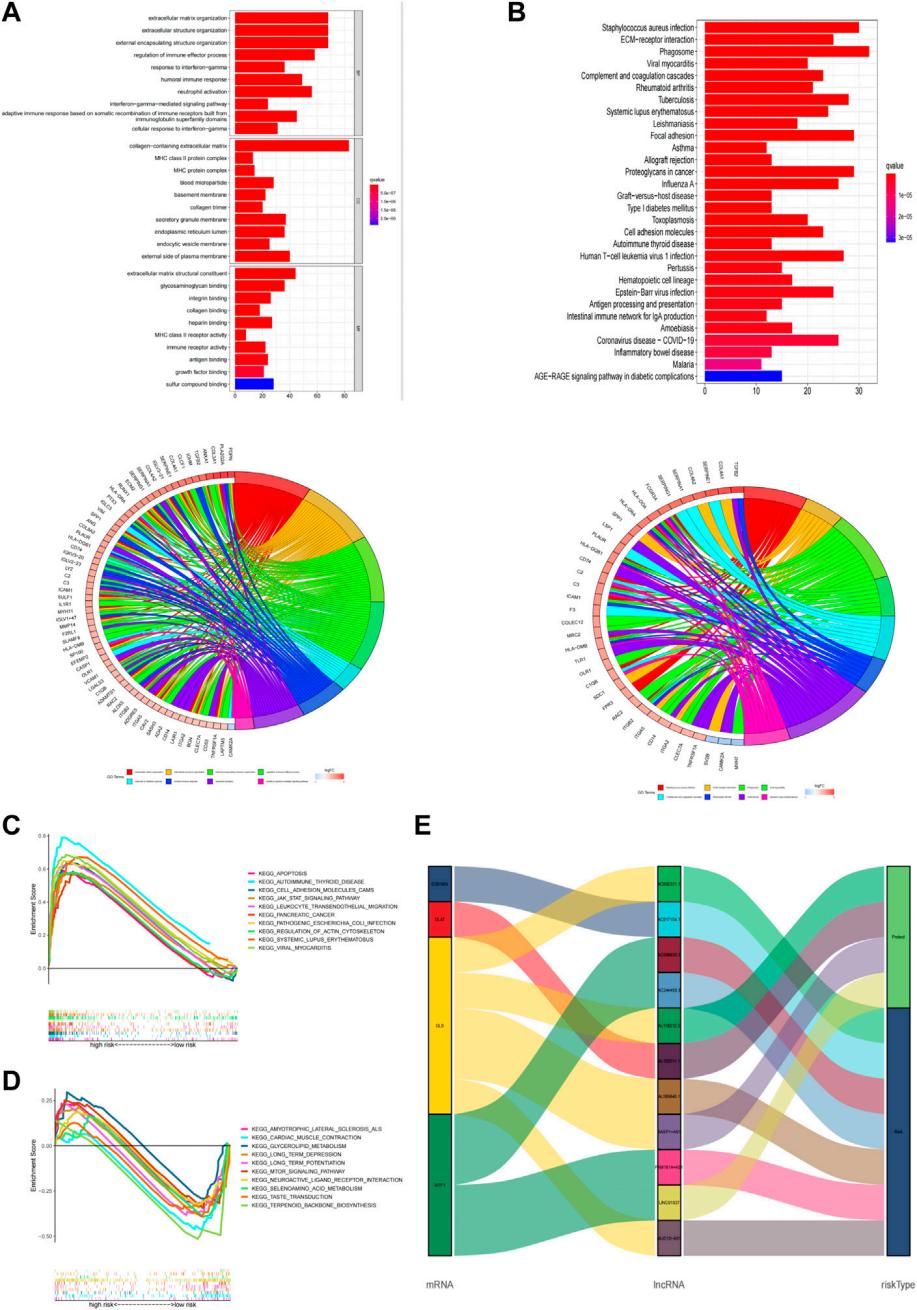
Functional analysis. **(A,B)** Top 10 classes of GO enrichment terms in biological process **(B)**, cellular component **(C)**, and molecular function (MF) based on 11 cuproptosis-related lncRNAs. **(C,D)** Top 30 classes of KEGG enrichment terms. **(E)** Sankey diagram.

GO and KEGG enrichment analysis was performed to explore potential bio-functions and signaling pathways of DEGs among high-risk and low-risk groups (Supplementary Table S6). According to the three dimensions of biological processes (BP), cellular components (CC), and molecular function (MF), we found that extracellular matrix organization, extracellular structure organization, and external encapsulating structure organization were the top three most BP-related functions in GO analysis (Figure 7A **,** Supplementary Table S7), while collagen−containing extracellular matrix, external side of the plasma membrane, and secretory granule membrane, as well as extracellular matrix structural constituent, glycosaminoglycan binding, and sulfur compound binding, were equally associated with CC and MF, respectively. Again, the pathways obtained using KEGG analysis (Figure 7B **,** Supplementary Table S7) mainly concerned processes such as phagosome, *staphylococcus aureus* infection, and focal adhesion. Additionally, we performed the GSEA analysis to compare the differences in signaling pathways enriched in the high- and low-risk groups based on the cuproptosis-associated lncRNA model (Supplementary Table S8). Apoptosis, autoimmune thyroid disease, and cell adhesion molecules cams were shown to be more associated with the high-risk group (Figure 7C), while amyotrophic lateral sclerosis als, cardiac muscle contraction, and glycerolipid metabolism were found to be more linked with the low-risk group (Figure 7D). And finally, using the Sankey diagram, a co-expression network between mRNA, modeling prognostic lncRNAs, and risk type was created to highlight their link with one another (Figure 7E).

### Composition and evaluation of tumor microenvironment

**FIGURE 8:**
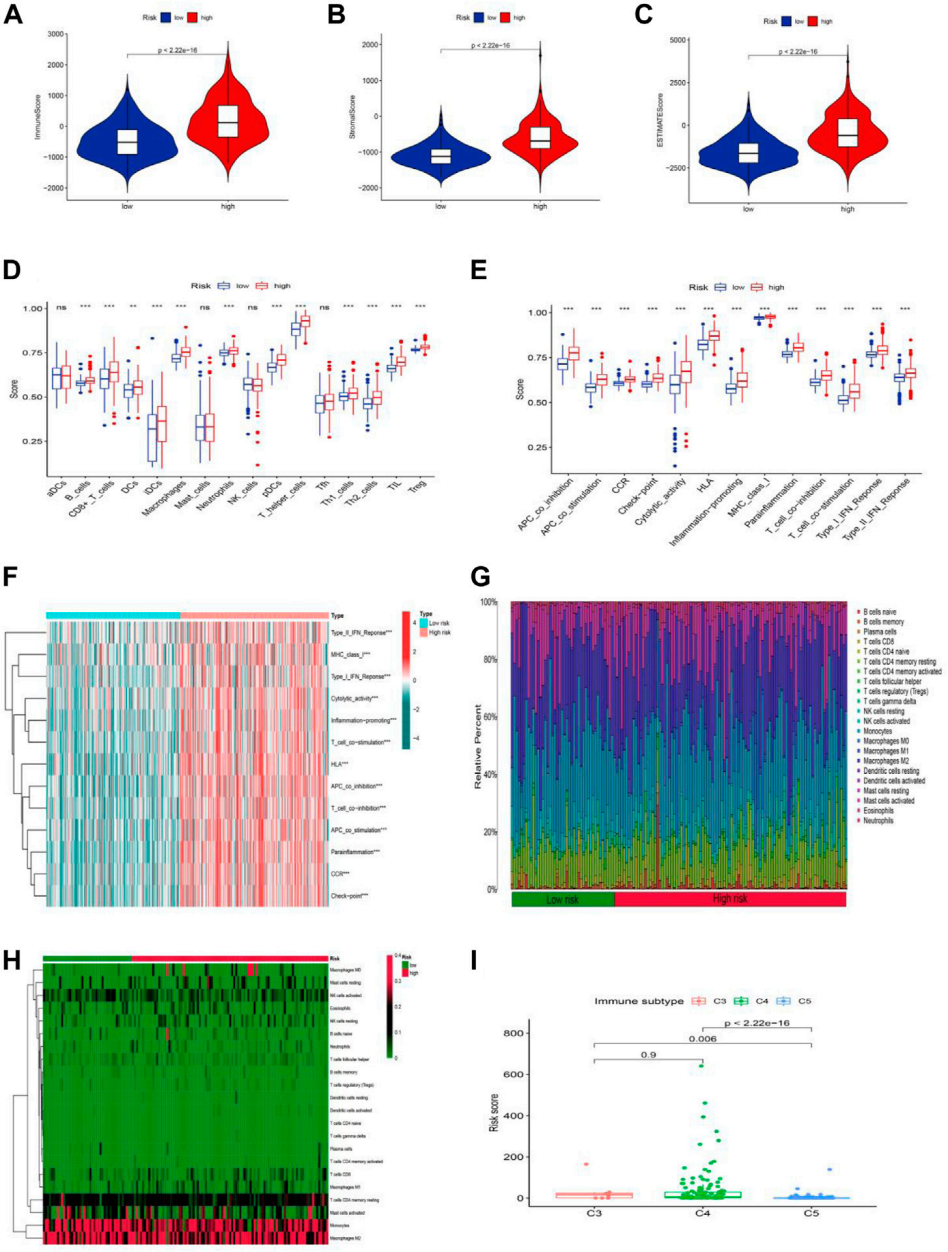
Stratification Analysis of the cuproptosis -related lncRNA prognostic risk score in immune features. **(A–C)** The assessment of TME related scores between high- and low-risk groups. **(D–F)** The score of immune cells comparing high-risk and low-risk groups by ssGSEA Score. **(G,H)** Heatmap of 22 tumor-infiltrating immune cell types in low- and high-risk groups. **(I)** LGG patients with different immune subtypes.

The tumor microenvironment is a promoter of tumor proliferation and inducer of immunological tolerance since it is the site of tumorigenesis and development, as well as a potential target for immunotherapy. Hence, the ESTIMATE algorithm was used to investigate the proportion of stromal and immune cells in TME in LGG. The results displayed in Figure 8A revealed that there were notably higher scores of immune, stromal, and ESTIMATE in the high-risk group compared to the low-risk groups, and we may infer from this that the high-risk group has more immune and stromal cells. In addition to this, we had also gone deeper into the composition of immune cells in tumor-infiltrating tissues according to the ssGSEA program (Supplementary Table S9). Except for a few immune cells like aDCs, mast, NK, and Tfh cells, most of the cells were in higher abundance in the high-risk group (Figure 8B). Surprisingly, all immune-related functions also appeared to be more relevant in the high-risk group than in the low-risk group (Figure 8C). The preceding findings suggested that patients with LGG in the high-risk group were more likely to respond to immunotherapy. We further applied the GSVA analysis to explore the underlying mechanism and immune pathways in two subgroups. The result is consistent with the above results presented that the high-risk group significantly connected immune pathways to the low-risk group, such as APC_co_inhibition, CCR, Check-point, MHC_class_I, Type_I_IFN_Reponse, and HLA (Figure 8D). To further compare the variance in immune cells between each patient in LGG, the CIBERSORT program was implemented to achieve this need. The proportion, infiltration capacity, and distribution of 22 immune cell types in the high- and low-risk groups were demonstrated by the box plot and heatmap (Figures 8E,F **,** Supplementary Table S10), respectively.

Yet the stability of the cuproptosis-associated lncRNA model on immunophenotyping was unclear, and we next evaluated the association between the risk score and the pre-published immune subtypes of multiple cancers to further corroborate the power of this risk signature for accurate immunophenotyping. As seen in Figure 8I, LGG patients with the C3 subtype exhibited a much higher risk score than that in C5 and C6 subtypes. We knew from earlier research that C3, C4, and C5 were inflammatory, lymphocyte-depleted, and immune-silent tumors, respectively, and that C3 was the type sensitive to immunotherapy in contrast to C4 and C5, offering a new insight based on the distinct features of this LGG immune microenvironment.

### Mutation analysis in LGG

**FIGURE 9:**
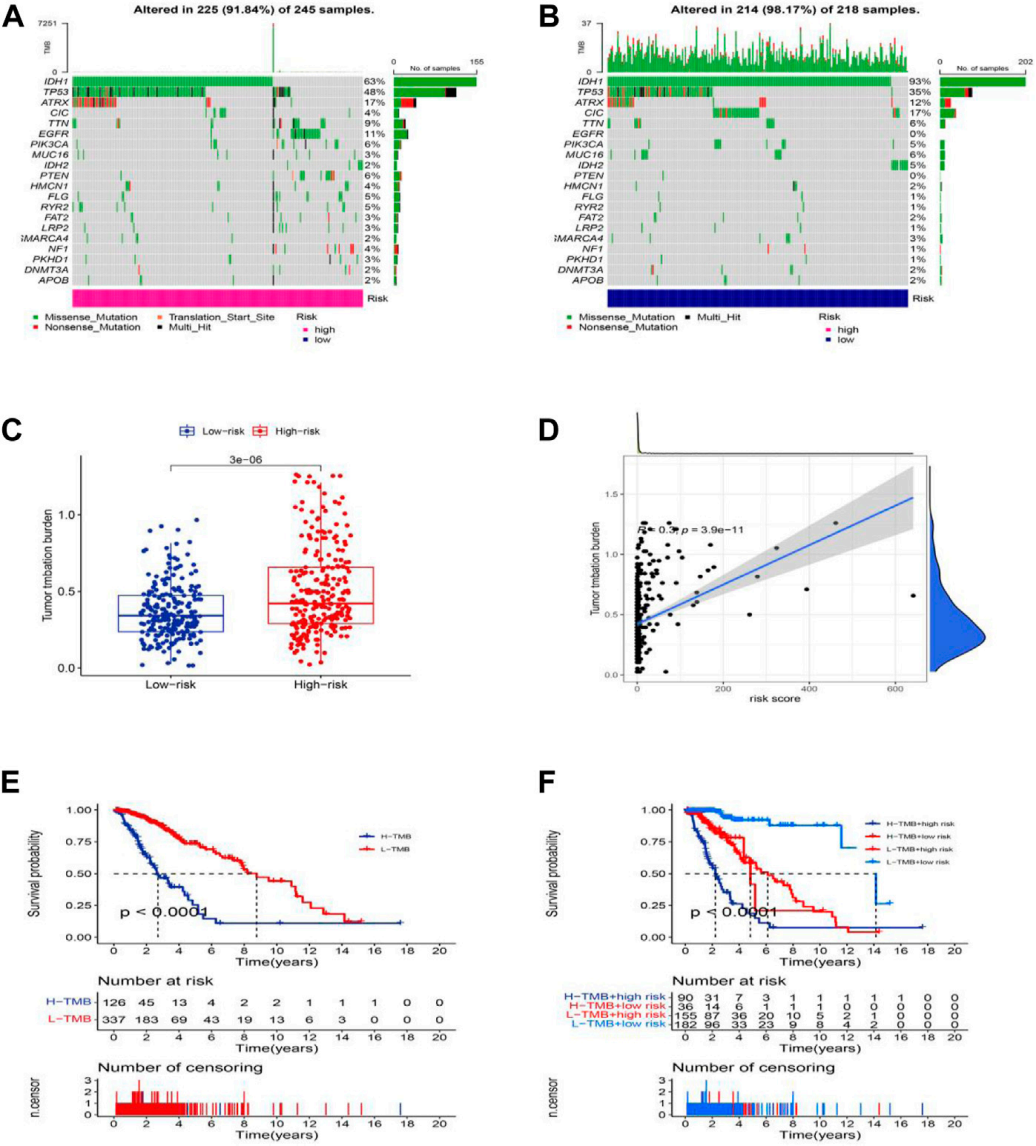
Somatic mutation landscapes in AML. **(A,B)** The top 20 mutation driving genes with the highest change frequency in the high-risk and low-risk groups. **(C)** Differences in TMB between high and low-risk groups. **(D)** Correlation between TMB and risk score. **(E)** Kaplan-Meier survival curves of the OS of patients in the high-TMB and low-TMB groups in the entire set. **(F)** Kaplan-Meier survival curves of the OS of patients based on the TMB and risk scores.

Moreover, we analyzed and integrated the mutation data and classified the mutations into different levels according to the mutation effect predictor. We present the top 20 driver genes that altered most frequently between the high-risk groups and low-risk groups in Figures 9A,B. Then, we scored TMB levels according to the somatic mutation data from TGCA. The TMB in the low-risk group was lower than that in the high-risk group (Figure 9C), further demonstrating that there was a significant correlation between the risk score based on the cuproptosis-related lncRNA and TMB (Figure 9D, R = 0.3, *p* = 3.9e-11). The survival of LGG patients in the high TMB group is better than that of the patients in the low TMB group. Additionally, the patients in the high-risk group with the high TMB group show a worse survival time, and the patients in the low-risk group with the low TMB group hold a significantly better OS (Figures 9E,F).

### Clinical treatment and drug sensitivity analysis

**FIGURE 10:**
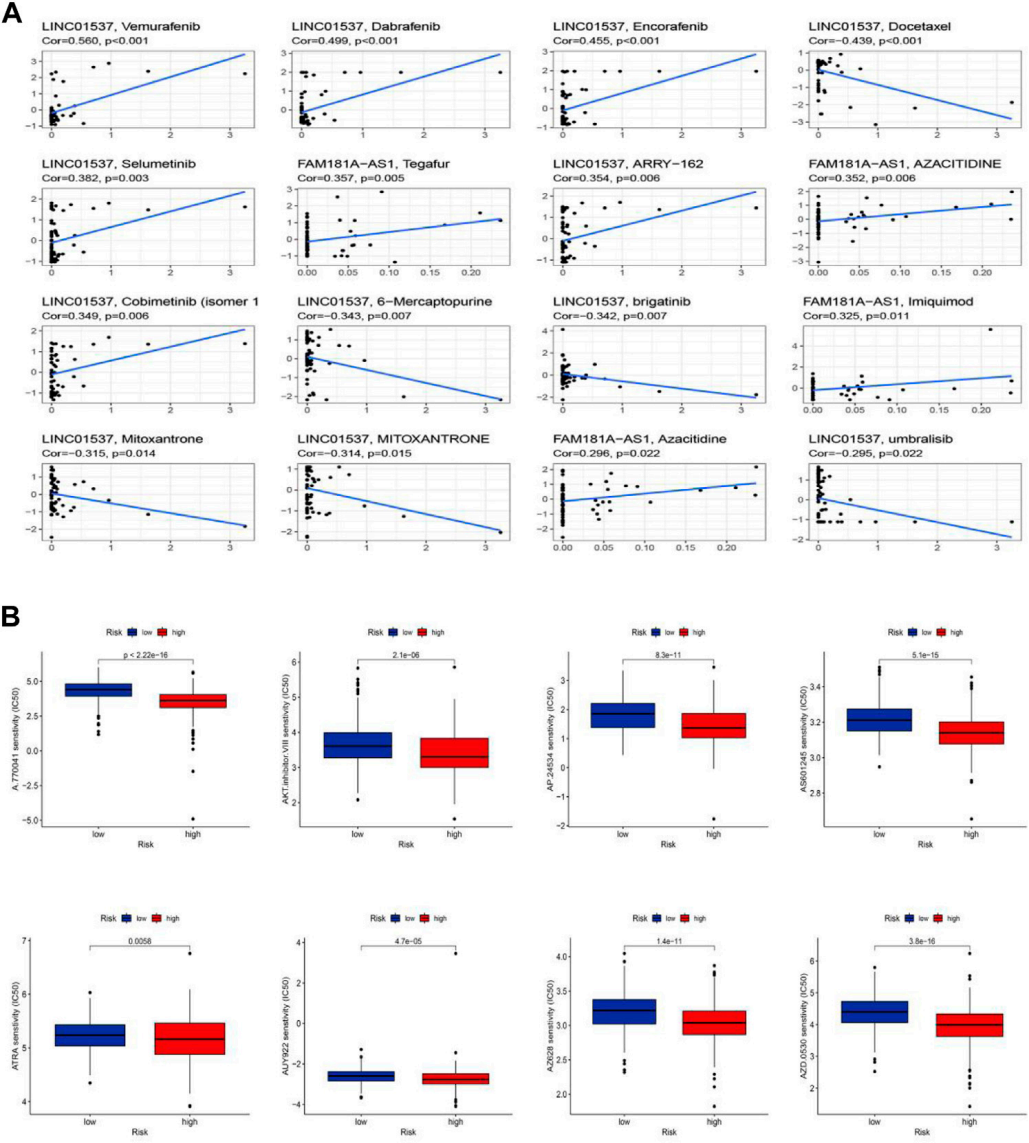
Sensitivity of chemotherapy drugs. **(A)** The high correlation drugs targeting 11 cuproptosis-related lncRNAs. **(B)** Drug sensitivity analysis.

We performed the drug sensitivity analysis to explore the high correlation drugs targeting 11 cuproptosis-related lncRNAs (Figure 10A, Supplementary Table S11). The results indicated that the correlation between drugs Vemurafenib and lncRNA LINC01537 was the highest (Cor = 0.56, *p* < 0.001), Dabrafenib, and lncRNA LINC01537 was the second (Cor = 0.499, *p* < 0.001), and so on. We speculated that the immunotherapy responses might be different between the low-risk and high-risk groups because of the significant differences in the immune microenvironment. To explore potential drugs targeting our risk model and improve treatments for patients with LGG, we used the IC50 values obtained from 138 patients using the “pRRophetic” package to determine therapeutic response. The IC50 of A.770041, AKT. inhibitor.VIII, AP.24534, AS601245, ATRA, AUY922, AZ628, and AZD.0530 were significantly higher in the low-risk group, suggesting that LGG patients in the high-risk group might benefit from those possible anti-tumor drugs (Figure 10B, *p* < 0.05). Thus, we may be able to select the most appropriate drugs for LGG patients, as well as our risk model based on the cuproptosis-related lncRNAs, which may provide a powerful cue for immunity therapy guidance.

### Validations for Long Non-coding RNAs expressions with quantitative real-time PCR

**FIGURE 11:**
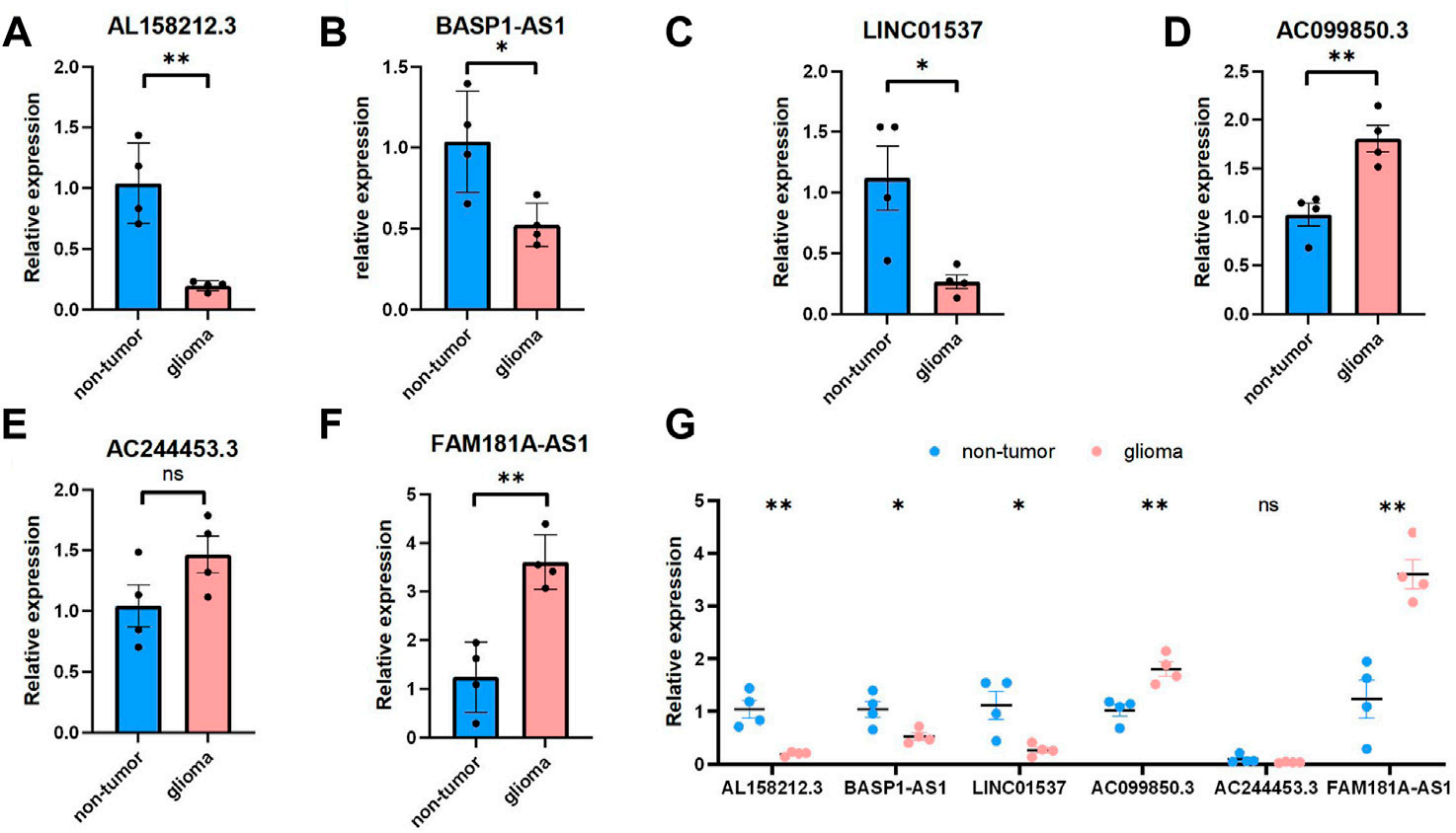
Validations for long non-coding RNAs expressions. **(A–C)** Expression analysis of three protective lncRNAs (AL158212.3, BASP1-AS1, LINC01537). **(D–F)** Expression analysis of three risky lncRNAs (AC099850.3, AC244453.3, FAM181A-AS1). **(G)** Expression levels of six Cuproptosis-related lncRNAs in the non-tumor and glioma by qPCR *p < 0.05, **p < 0.01, ***p < 0.001, and ns No significance.

We selected six Cuproptosis-related lncRNAs for validation, and we detected their expression levels in non-tumor brain tissues and glioma tissues, By using RT-qPCR assay, the expression level of AL158212.3,BASP1-AS1, LINC01537 showed an overall upward trend in non-tumor brain tissues, the expression level of AC099850.3,AC244453.3,FAM181A-AS1 showed an overall upward trend in glioma tissues. However, through further verification, there is no significant difference in the expression level of AC244453.3 between non-tumor tissues and LGG, which may be due to the small number of samples (Figure 11).

## Discussion

LGG is heterogeneous and highly aggressive, and patient treatment and prognosis prediction are the focus (Ostrom et al., 2018). However, studies have shown that the prognosis of LGG cannot be completely predicted based on histological grading and currently clinically available molecular biomarkers (Louis et al., 2016). Tumor cells have abnormal cell death, and molecular markers for predicting cell death can effectively predict overall cancer survival (Vogler et al., 2009). Regulated cell death (RCD), including apoptosis, entosis, necroptosis, pyroptosis, and ferroptosis, plays an important role in anticancer mechanisms (Koren and Fuchs, 2021). For example, Meike and his team found that proper regulation of BCL2A1 and BCL-XL could improve the resistance of leukemia cells to therapeutic agents (Vogler et al., 2009). RIPK3 plays an important role in the regulation of necroptosis in breast and colorectal cancers (Vergara et al., 2020). At the same time, in LGG, Guilherme and the team also found that RIPK3 is an independent prognostic marker of LGG, and the combination of RIPK3 level and IDH mutation status can improve the overall survival rate of LGG patients (Vergara et al., 2020). Since the concept of ferroptosis was proposed, ferroptosis has become a research hotspot in RCD. Wang and his team found that P53 acetylation can enhance ferroptosis and achieve the effect of inhibiting tumors (Wang et al., 2016). In addition, the presence of enzymes that control ferroptosis, such as GPX4, also enables targeted approaches to target ferroptosis to eliminate cancer (Yang et al., 2014). Cuproptosis has recently been shown to play a role in RDC as well. Tsvetkov and others demonstrated that intracellular copper accumulation induces aggregation of mitochondrial fatty acylated proteins, reducing the stability of Fe-S cluster proteins and leading to cell death (Tsvetkov et al., 2022). Similarly, the study also found that ferredoxin 1 (FDX1) has a positive effect on cuproptosis, and buthionine sulfoximine (BSO) has a certain role in the cuproptosis mechanism of lung cancer cells. These biomarkers and the mechanisms within them may help with the application of cuproptosis in cancer treatment and prediction.

In recent years, more and more researchers have discovered that long non-coding RNAs (LncRNAs) play an important role in the progression and metastasis of malignant tumors (Liang et al., 2020). Moreover, the study of lncRNAs in LGG is gradually increasing. LINC00941 and BASP1-AS1, as hypoxia-related lncRNAs (HRLs), have been found to affect LGG proliferation in related studies and can be used as novel biomarkers to predict the prognosis and potential therapeutic targets of LGG patients (Xu et al., 2021). Kang and his team also found that the expression levels of H19, LINC02587, AC015909.3, and others can be used as lncRNAs risk models to help identify patients who are suitable for related treatments and improve the status of LGG treatment (Kang et al., 2021). However, given that the cuproptosis mechanism was recently discovered, the relationship between cuproptosis and lncRNAs is largely unknown. Here, we established a model of lncRNAs associated with cuproptosis to explore their relationship and predict the prognosis of LGG patients.

In our study, we identified 1149 cuproptosis-related lncRNAs, of which 11 cuproptosis-related lncRNAs were screened as OS prediction models in LGG patients. Risk signature screening was performed for seven risk factors (MUC12-AS1 AL589843.1 AC099850.3 AC017104.1 AC244453.3 FAM181A-AS1 AC002351.1) and four protective factors (AL158212.3 BASP1-AS1 AL162511.1 LINC01537). Some of these lncRNAs appeared in the construction of prognostic prediction models. For example, BASP1-AS1 is positively correlated with the prognosis of LGG and may be a target for LGG therapy (Xu et al., 2021). Jiang and others demonstrated that FAM181A-AS1 could promote gliomagenesis by sponging miR-129–5p (Jiang and Chen, 2020). As ferroptosis-related lncRNAs, LINC01537 can be used as a prognostic predictor for lung adenocarcinoma (LUAD), with potential implications for the treatment of LUAD patients (Lu et al., 2021). AL589843.1 was shown to be a prognostic feature of bladder cancer (Zheng et al., 2021). AC099850.3 promotes the proliferation and invasion of HCC through the PRR11/PI3K/AKT axis and is associated with patient prognosis (Wu et al., 2021).

When we screened drug candidates by assessing the response of the samples against the GDSC database, we were pleasantly surprised to find that the low-risk group was more resistant to all compounds, implying that high-risk patients may have a higher susceptibility to these drugs. Immunotherapy is promising in the treatment of brain tumors, and predictive biomarkers have been shown to play a role in the immunotherapy of tumor patients. For example, the oncogenic activation pathway PI3K-AKT-mTOR pathway was shown to induce ferroptosis in LGG cells; This can help predict disease outcome and response to treatment (Lin and Okada, 2016). In a study of colorectal cancer patients, Lin et al. also found that immune checkpoint inhibitors (ICIs) have a better role in the treatment of cancer patients, and specific predictive markers can improve the clinical response differences of different patients. To better improve the prognosis of colorectal cancer (Lin et al., 2020). The immune landscape analysis of cuproptosis-related lncRNAs in this study showed that there were significant differences between the high and low-risk groups. The low-risk group had lower tumor purity and higher scores than the high-risk group. After evaluating the immune markers of LGG patients, we found that the immune infiltration of iDCs, pDCs, and T helper cells in the low-risk group was significantly reduced. Rhee et al. also found that the lower the tumor purity, the higher the immune cell infiltration, and the higher the expression of specific cell genes, which was negatively correlated (Rhee et al., 2018). Likewise, Jurjen and co-workers found that human pDCs act as potent activators of CD8 (+) T in antitumor responses with significant anticancer effects (Tel et al., 2013). In addition, Victoria et al. in the study of activating iDCs, and Pornpimon et al. in the study of IL-9-producing T helper cells, found that the growth of iDCs and T helper cells can significantly inhibit the growth of cancer (Jennings et al., 2019; Angkasekwinai and Dong, 2021). This is consistent with our GO analysis results. In addition, according to GSEA analysis, the high-risk group was significantly enriched in immune response and metabolism-related pathways.

The number of coding-competent bases subject to somatic mutation is called TMB. TMB can induce the formation of new antigens, thereby triggering antitumor immunity. TMB can serve as an effective biomarker for predicting response to immunotherapy (Gandara et al., 2018). In our study, the low-risk group had lower TMB than the high-risk group, indicating that the high-risk group responded better to immunotherapy. This may be related to the fact that high TMB favors the infiltration of Tregs in KIRC, CD8^+^ T cells, and macrophages in LGG (Li et al., 2021). As mentioned above, we concluded that the high infiltration of iDCs, pDCs, and T helper cells in LGG and TMB levels in high-risk groups in TMB analysis might provide new directions for guiding the treatment of LGG, and our model may be able to become an Immune biomarker in LGG patients. In addition, when TMB levels were scored according to the somatic mutation data in TGCA, the results showed that TMB in the low-risk group was lower than that in the high-risk group. We speculate that indicators based on cuproptosis are highly correlated with TMB, and since the low-risk group patients have an immune function, we speculate that there may be a correlation between immune escape and cuproptosis to improve prognosis, and this model provides new insights for us to better understand the role of cuproptosis-related lncRNAs in LGG. However, more research is needed to prove it.

From a clinical perspective, the pathological stage can be a decisive factor in the prognosis of LGG patients. However, due to the present periodization, systems are incompetent to accurately predict the prognosis of LGG patients. LGG patients always have different clinical outcomes irrespective of the same stage. So, novel predictive biomarkers are urgently needed. Here, we established a cuproptosis-related lncRNAs model that can predict the prognosis of LGG patients. Our study also offers insights for further understanding the mechanism of cuproptosis. We utilized multiple methods to support the reliability of the model and optimized it. We assumed that the model was dependable without external data validation. We do aware that there are some limitations in this study. First, additional clinical datasets are required to validate the model. Second, the molecular biological function of cuproptosis-related lncRNAs has not been fully explored. Thus, we’d like to recollect clinical samples to further confirm our study. Besides, we will perform a further study to explore the molecular biology functional mechanism of cuproptosis-related lncRNAs in LGG.

In conclusion, we constructed a prognostic model that is sensitive and reliable, and it showed well efficacy in predicting the prognosis of patients with LGG. Additionally, the model can also predict the response to immunotherapies of LGG patients. Subsequently, we screen potential drugs to improve current treatment management. On the other hand, our study laid the groundwork for exploring the role of cuproptosis in the biogenesis and progression of LGG.

## Data Availability

The original contributions presented in the study are included in the article/supplementary material, further inquiries can be directed to the corresponding author/s.
